# Oxidant balance in brain of rats receiving different compounds of selenium

**DOI:** 10.1007/s10534-013-9654-y

**Published:** 2013-07-10

**Authors:** Irena Musik, Małgorzata Kiełczykowska, Joanna Kocot

**Affiliations:** Chair and Department of Medical Chemistry, Medical University of Lublin, Chodźki 4a, 20-093 Lublin, Poland

**Keywords:** Selenium, Brain, Antioxidant defence, Lipid peroxidation, Male rats

## Abstract

The influence of two organic selenocompounds and sodium selenite on oxidant processes in rat brain tissue was investigated. The study was performed on male Wistar rats. The animals were divided into four groups: I—control; II—administered with sodium selenite; III—provided with selenoorganic compound A of chain structure 4-(*o*-tolyl-)-selenosemicarbazide of 2-chlorobenzoic acid and IV—provided with selenoorganic compound B of ring structure 3-(2-chlorobenzoylamino-)-2-(*o*-tolylimino-)-4-methyl-4-selenazoline. Rats were treated by stomach tube at a dose of 5 × 10^−4^ mg of selenium/g of b.w. once a day for a period of 10 days. In brain homogenates total antioxidant status (TAS), activities of superoxide dismutase (SOD) and glutathione peroxidase (GPx), concentrations of ascorbic acid (AA) and reduced glutathione (GSH) as well as concentration of malonyl dialdehyde (MDA) were determined. TAS was insignificantly diminished in all selenium-supplemented groups versus control. SOD was not significantly influenced by administration of selenium. GPx was markedly decreased in group III versus control, whereas increased in group IV versus control and group III. Selenosemicarbazide depleted AA in well-marked way versus group II. GSH was significantly depressed in group III versus both control and group II and diminished in group IV versus group II. MDA was significantly decreased in group III versus both control and group II, whereas in group IV increased versus group III. As selenazoline A did not decrease elements of antioxidant barrier and increased GPx activity, it seems to be a promising agent for future studies concerning its possible application as a selenium supplement.

## Introduction

Selenium is an essential trace element, necessary to correct organism’s functions (Yan et al. 2013). Its deficit may result in severe disorders including cancer (Klarod et al. 2011), dermatic and mood disorders (Ingen-Housz-Oro et al. 2004; Sopjani et al. 2008), illnesses of alimentary tract (Skelton et al. 2006), as well as AIDS development after HIV-infection (Rayman 2000). Selenium is also suggested to be involved into functioning of cartilage (Yan et al. 2013). Numerous research studies precede our studies on safe and effective supplements (Combs 2005; Řezanka and Sigler 2008; Selamoglu Talas et al. 2009) using diverse substances both inorganic (sodium selenite or selenate) (Ivancic and Weiss 2001; Ayaz and Turan 2006; Uezono et al. 2006) and organic (Xia et al. 2004; Burk et al. 2006; Pawlas and Małecki 2007; Cui et al. 2008; Selamoglu Talas et al. 2009) as well as selenium-enriched natural products e.g.: malt (Liu et al. 2006), yeast (Burk et al. 2006), broccoli (Řezanka and Sigler 2008), but the question of the best form remains unsolved. The main difficulties result from the narrow range between therapeutic and toxic dose of selenium (Hawkes et al. 2008) as well as from the dependence of its bioavailability on the form of supplementation (Burk et al. 2006). Since selenium is considered to be an antioxidant, as a constituent of one of the main antioxidative enzymes—glutathione peroxidase (Ha and Smith 2009), many investigations have concerned relationships between selenium and oxidative balance in organisms (Ghodbane et al. 2011; Horky et al. 2012; Zhang et al. 2013). Ebselen, a ring selenoorganic compound of isoselenazole structure has been found to possess antioxidant properties although its negative effects have also been stated (Farina et al. 2004; Shi et al. 2006). An organoselenium compound has also been shown to exert protective effect against side effects of cisplatin by the affecting of pro- and antioxidative processes (Ghosh et al. 2013).

Selenium is widely distributed throughout the body, and its high level occurs in the brain (van Eersel et al. 2010). As an antioxidant or a main constituent of brain selenoproteins, it appears to be an important factor in maintaining of brain functions (Akbaraly et al. 2007). Associations between low selenium levels and a significantly greater incidence of depression and other negative mood states such as anxiety, confusion, and hostility (Rayman 2000) as well as cognitive impairment, Alzheimer’s disease (van Eersel et al. 2010) or brain tumors (Chen and Berry 2003) have been reported. Researchers have indicated that neurological disorders are often associated with oxidative stress (Chauhan et al. 2004; Mariani et al. 2005) and selenium compounds may display antioxidant and neuroprotective properties (Akbaraly et al. 2007). Having regarded these findings the aim of the present study was to evaluate the influence of the two newly synthesized organic selenocompounds with that exerted by acknowledged inorganic supplement sodium selenite, which is still used in clinical practice (Pagmantidis et al. 2008; Schnabel et al. 2008; Savory et al. 2012) and as a supplement of animal food (Pavlović et al. 2010), on oxidant processes in rat brain tissue.

## Materials and methods

### Chemicals

Two selenoorganic compounds were synthesized in our chair: compound A (chain structure) 4-(*o*-tolyl-)-selenosemicarbazide of 2-chlorobenzoic acid (Musik et al. 2002) and compound B (ring structure) 3-(2-chlorobenzoylamino-)-2-(*o*-tolylimino-)-4-methyl-4-selenazoline (Musik et al. 2009). 

### Animal experiment

The experiment was carried out on adolescent male Wistar rats. After 3 days of acclimatization the animals were randomly divided into four groups (ten animals each): group I (control with no selenium supplementation)—treated with saline, group II—treated with sodium selenite, group III—treated with 4-(*o*-tolyl-)-selenosemicarbazide of 2-chlorobenzoic acid, group IV—treated with 3-(2-chlorobenzoylamino-)-2-(*o*-tolylimino-)-4-methyl-4-selenazoline.

At the beginning of the experiment the weights of rats were included within a range of 110–150 g. Sodium selenite was given in form of water solution. Organic compounds A and B given to groups III and IV were suspended in the emulsion composed of oil, Arabic gum and water in the following proportion 2:1:1.5. The administration was performed by stomach tube. Selenium compounds were given to rats at a dose of 5 × 10^−4^ mg of selenium/g of b.w. once a day for a period of 10 days. Body weights of animals were measured every day before selenium administration and the appropriate amount of selenium compound was calculated for each animal. The administered dose was relatively high but it was applied with the aim of studying toxicity of the new organoselenium compounds. It is difficult to foresee the toxic dose of a new compound as the bioavailability depends on its structure. The dose was chosen considering those applied by other authors which were included in the range from 1 × 10^−4^ mg/g b.w. (Medeiros et al. 2012) to 19.7 × 10^−4^ mg/g b.w. (Selamoglu Talas et al. 2008), mainly 2 × 10^−4^–3 × 10^−4^ mg/g b.w. (El-Demerdash 2004; Agarval and Behari 2007; Naziroğlu et al. 2008; Akil et al. 2011a). Rats had free access to standard feed containing adequate level of selenium and drinking water. After the end of the experiment animals were sacrificed under pentothal narcosis.

The study was performed according to statutory bioethical standards and approved by I Local Ethical Commission of Medical University of Lublin, acceptance no. 65/AM/2004.

### Preparing of brain samples and measurement of biochemical parameters

The samples of brain tissue were collected. Ten per cent (w/v) tissue homogenates were prepared in 0.1 mol/dm^3^ Tris–HCl buffer, pH = 7.4. Supernatants were obtained by centrifugation at 5,000×*g* for 30 min. The prepared material was stored at temperature −18 °C.

The following substances were determined in brain homogenates: total antioxidant status (TAS), activity of antioxidant enzymes—superoxide dismutase (SOD) and glutathione peroxidase (GPx), concentrations of non-enzymatic antioxidants—ascorbic acid (AA) and reduced glutathione (GSH) as well as concentration of lipid peroxidation marker—malonyl dialdehyde (MDA). TAS was measured using diagnostic kit produced by RANDOX and expressed in mmol/g of protein. SOD and GPx activities were determined using diagnostic kits RANSOD and RANSEL produced by RANDOX and expressed in U/mg of protein and U/g of protein, respectively. GSH concentration was determined using BIOXYTECH^®^ GSH-400™ kit produced by OxisResearch™ and expressed in μg of GSH/mg of protein. AA concentration was determined using modified Kyaw method (Rutkowski and Grzegorczyk 1998) and expressed in μmol of AA/g of protein. MDA concentration was determined using Ledwożyw et al. (1986) method and expressed in nmol of MDA/mg of protein. Protein was measured using method of Bradford (1976). The assays were performed with use of spectrophotometer SPECORD M40 (Zeiss Jena).

### Statistical analysis

Statistical analysis was performed using ANOVA test. Comparisons between control and selenium-supplemented groups as well as between selenium-supplemented groups were made using the Tukey’s HSD test or Dunnett’s T3 test. Values were considered significant with *p* < 0.05. The choice of multiple comparisons test was dependent on evaluation of variance homogeneity in compared groups which was performed using Leven’s test. In the event of homogeneous variances Tukey’s method of honestly significant differences—HSD test was used, whereas when variances in groups were heterogeneous (*p* < 0.05 in Leven’s test) T3 Dunnet’s test (applicable in the case of heterogenous variances) was applied.

## Results

TAS values were diminished in all selenium-supplemented groups, although this effect was not significant versus control with no selenium supplementation.

Activities of antioxidant enzymes GPx and SOD were slightly increased versus control group in group II (inorganic sodium selenite). In group III (organic chain selenocompound) SOD was unchanged, whereas GPx was markedly decreased versus control. In group IV (organic ring selenocompound) both enzymes were increased versus control, although this effect was significant only in the case of GPx. In the case of GPx organic forms of selenium displayed insignificant differences when compared to inorganic selenite—in group III (chain compound) GPx was decreased versus group II, whereas in group IV (ring derivative) enhancement versus group II was observed. As concerns comparison between two studied organic forms—the significant increase in group IV versus group III was also observed.

Brain low-molecular antioxidants, AA and GSH were influenced by selenium-administration in different way, depending on the form of supplements. All the studied selenium compounds insignificantly decreased AA concentration versus control, whereas chain selenosemicarbazide depleted it in well-marked way versus group II (selenite). GSH was slightly enhanced in group II, significantly depressed in group III and practically unchanged in IV one in comparison with control. Both selenoorganic compounds significantly decreased GSH concentration in comparison with inorganic selenite group.

MDA brain concentration was unaltered in rats of group II (inorganic sodium selenite), significantly decreased in group III and insignificantly increased in group IV versus control. Organic forms of selenium showed differences compared to inorganic selenite—in group III (chain compound) well-marked decrease in MDA versus group II, whereas in group IV (ring selenocompound) slight enhancement versus group II were observed. Moreover, there was the significant increase in MDA in group IV versus group III.

**Fig. 1:**
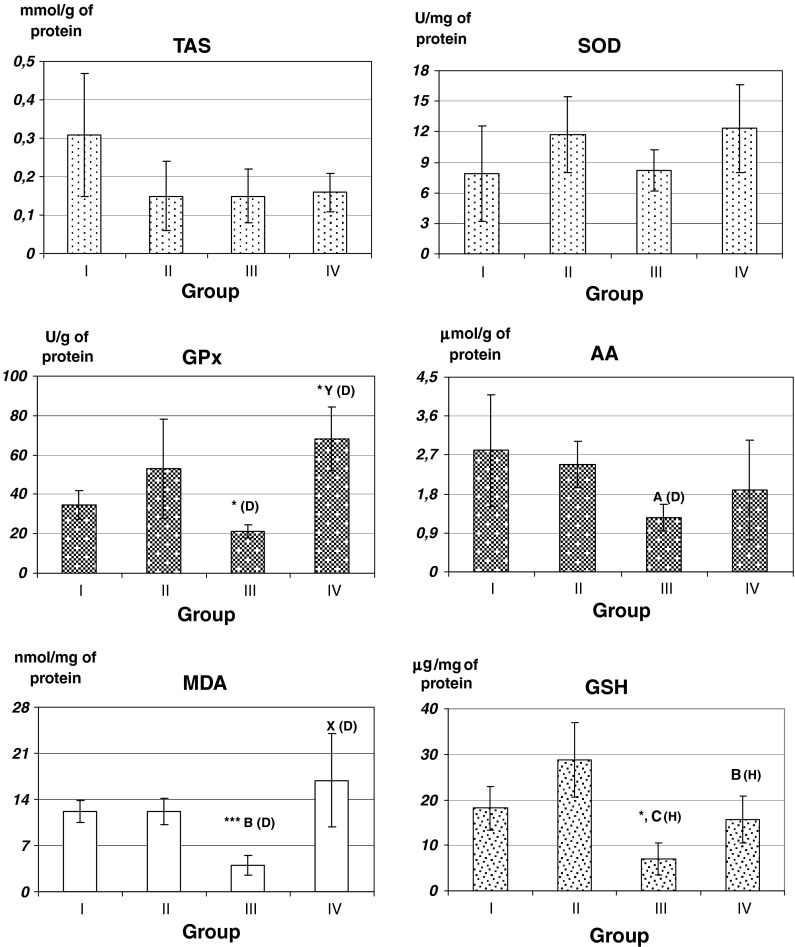
Effect of selenium supplementation on oxidative parameters in brain of rats. Rats were randomly divided into four groups (10 animals each) and intragastrically treated with: saline (group I); sodium selenite (group II), 4-(*o*-tolyl-)-selenosemicarbazide of 2-chlorobenzoic acid (group III) and 3-(2-chlorobenzoylamino-)-2-(*o*-tolylimino-)-4-methyl-4-selenazoline (group IV). Data are means ± SD. *p < 0.05; ***p < 0.001 versus group I ^A^ *p* < 0.05; ^B^ *p* < 0.01; ^C^ *p* < 0.001 versus group II ^X^ *p* < 0.05; ^Y^ *p* < 0.01; versus group III (H) Tukey’s HSD test (D) T3 Dunnett’s test

All the obtained results are presented in Fig. 1.

## Discussion

Selenium is an essential element and its deficit may result in occurring of diverse disorders of organism functions. Selenium supplementation has been found to show beneficial effects in different pathological states. On the other hand supplementation must be carried out taking precautions as this element can cause negative effects, including disturbances of nervous system. Among other things, selenite was found to cause irregular neurons growth in fish embryos (Ma et al. 2012).

In the present study TAS was insignificantly decreased in selenium-administered rats. The lack of distinct relationships between selenium and TAS was also reported by Okuonghae et al. (2011) who found that in rats exposed to premium motor spirit fumes well-marked increase in plasma selenium was accompanied with no significant changes of TAS value. Another study also revealed no significant correlations between selenium supplementation and blood TAS (Savory et al. 2012). The decrease in TAS value observed in our study can hardly be connected with the changes of any other studied antioxidant. As distinct from TAS changes these alterations depended on the structure on the used selenocompound. It is possible that TAS decrease could be related with other substances of antioxidant properties. Among other things some selenoproteins (selenoprotein P, thioredoxin reductases and methionine sulphoxide reductase B) were found to act as antioxidants (Hoffmann et al. 2007; Steibrenner and Sies 2013). Further researches including the influence of selenium supplementation on expression of these proteins might contribute to the clarification of this question. When considering reported opinions on selenium supplementation and our results (Herrmann et al. 2001; Feng et al. 2012), measuring TAS is more recommended than determining changings of individual antioxidants.

Antioxidative enzymes GPx and SOD were increased by inorganic selenium and selenoorganic ring compound, whereas the chain derivative caused their decrease versus control, although these effects were significant only in the case of GPx in groups III and IV (selenoorganic compounds). The outcomes of the present study concerning sodium selenite administration are confirmed by other authors’ reports. Sodium selenite increased SOD and GPx activity in brain of cadmium-exposed as well as non-exposed suckling rats. The applied dose and period of treatment were comparable—0.632 mg selenium/kg b.w. and 9 days, respectively (Lazarus et al. 2011) to those used in the present experiment. In mice treated with methylmercury GPx activity in cerebral cortex was decreased and co-administration of sodium selenite did not exert any influence, whereas in non-exposed animals increase in GPx was observed (Glaser et al. 2010). Selenium given as sodium selenite increased GPx activity in cerebrum and cerebellum of suckling rats whose mother were administered methimazole during pregnancy and lactation. The same significant enhancement was observed in animals with no methimazole treatment. In the case of SOD increase was shown only in cerebellum of animals treated with methimazole, whereas in untreated selenium displayed no effect (Ben Amara et al. 2009).

As far as selenoorganic compounds are concerned the outcomes of the present study and others are divergent than what seems to be connected with different structures of the administered selenocompounds. Administration of dl-selenomethionine to mercury-exposed rat pups whose mothers were also subjected to the same treatment during pregnancy resulted in significant increase in hippocampal SOD activity (Su et al. 2008). Medeiros et al. (2012) found that selenoorganic compound of chain structure increased SOD activity only in the rat hippocampus, whereas GPx was reduced in all studied brain structures (cerebral cortex, hippocampus and cerebellum). In the present experiment the same result was obtained with regard to GPx in group III receiving chain selenocompound. Different results were obtained by Selamoglu Talas et al. In rats exposed to carcinogenic hydrocarbon, selenium, administered in form of two selenones, caused increase in both studied enzymes’ activity (Selamoglu Talas et al. 2008). The differences between the referred findings and ours might result from the fact that in the present work the period of selenium administration was shorter. This assumption seems to be confirmed by observations reported by Ozkan et al. (2007) who found that in mice, exposed to cigarette smoke and selenium in form of l-selenomethionine, no significant changes of SOD and GPx were observed after 3 months, whereas after 5 months both enzymes were found to be increased. The significant decrease in GPx observed in the present study in group III could be caused by the comparatively great dose, whereas no changes or increase reported by Ozkan et al. may result from adaptive mechanism developed by organism during longer time of exposure.

Low-molecular antioxidants AA and GSH were not markedly influenced versus control in the present study, except for GSH in group III. Similarly, Agarval and Behari (2007) found that sodium selenite did not influence GSH concentration in brain of healthy and mercury-exposed rats. Sodium selenite showed ameliorating effect consisting in significant increase in GSH as well as in vitamin C concentration in cerebrum and cerebellum of rats undergoing exposure to chromium, whereas in non-exposed animals no significant effects were observed (Soudani et al. 2012), which confirms outcomes of the present study. Other scientists reported different outcomes but their experiments concerned selenium influence in animals additionally exposed to pathogenic factors. Naziroğlu et al. (2008) reported that in rats with pentylentetrazol-induced seizures earlier selenium administration resulted in both GSH and AA increase in cortex brain. As for GSH other studies were consistent with findings reported by Naziroğlu et al. In mercury-exposed rat pups whose mothers were also subjected to the same treatment during pregnancy dl-selenomethionine caused significant increase in hippocampal GSH concentration (Su et al. 2008). Increase in GSH concentration in rats exposed to carcinogenic hydrocarbon and receiving two selenooorganic compounds (selenones) was also observed by Selamoglu Talas et al. (2008). Selenium given as sodium selenite increased GSH concentration in cerebrum and cerebellum of suckling rats whose mothers were treated with methimazole during pregnancy and lactation. The similar significant enhancement was observed in animals with no methimazole treatment (Ben Amara et al. 2009).

In the present study MDA, a marker of lipid peroxidation, was not significantly influenced versus control, except for group III. The studies performed by other researchers are consistent with our results concerning inorganic selenite. In brain of healthy and mercury-exposed rats sodium selenite did not change MDA concentration (Agarval and Behari 2007). The similar effects were reported by Akil et al. (2011a, b) who stated no effect of selenium supplementation (sodium selenite at two different doses: 6 mg/kg/day and 600 μg/kg/day) on MDA values in brain tissue and blood samples, respectively. Sodium selenite did not affect MDA concentration in cerebrum and cerebellum of suckling rats whose mothers were treated with methimazole during pregnancy and lactation. The same lack of influence was displayed in animals without methimazole administration (Ben Amara et al. 2009). In some cases of additional poisoning with heavy metals the outcomes of other authors were different. Sodium selenite given to rats did not influence distinctly brain TBARS (thiobarbituric acid-reactive substances) level in healthy animals, but in those exposed to aluminium significant decrease was observed (El-Demerdash 2004). Sodium selenite depressed TBARS in brain of suckling rats exposed to cadmium, whereas in non-exposed no significant influence was obtained (Lazarus et al. 2011). Similarly, sodium selenite exerted ameliorating effects on lipid peroxidation induced in rat cerebrum and cerebellum by chromium intoxication. MDA concentration was found to be significantly decreased. In non-exposed rats no influence was observed (Soudani et al. 2012).

The results concerning organic selenium are not consistent. It can be connected with the fact that administered compounds had different structures. Differences could also be the consequences of the additional pathogenic factors to which animals were exposed. Hippocampal MDA concentration in rat pups exposed to mercury and receiving selenium in form of dl-selenomethionine, whose mothers were also subjected to the same treatment during pregnancy, was significantly increased (Su et al. 2008). Administration of selenium, in form of two organic compounds (selenones), to rats exposed to carcinogenic hydrocarbon resulted in distinct decrease in MDA concentration (Selamoglu Talas et al. 2008). Organoselenium compound of chain structure did not influence in well-marked way TBARS in rat hippocampus and cerebellum, whereas enhancement in the cerebral cortex was observed (Medeiros et al. 2012). Insignificant increase was observed in the present work in group IV, but the time of administration was considerably shorter (10 vs. 30 days).

Interestingly, in the present study the changes of GPx activity and GSH concentration were practically the same in groups II and III. In vitro study performed on cortical neurons of rat fetuses treated with organoselenium ring compound (ebselen) showed that concentration of GSH increased up to specific value of ebselen concentration. Further enhancement of ebselen concentration did not influence GSH concentration (Pawlas and Małecki 2007). Venardos et al. (2004) observed the similar dependence between liver GPx activity and selenium level in diet of experimental animals. It points that GSH, similarly as GPx activity, increases in selenium-dose-dependent way till a kind of “saturation” occurs. These observations are consistent, considering the fact that GSH is a substrate for GPx.

Selenium supplementation was found to show beneficial effect in cases of disturbances of nervous system. The study performed on rats revealed that selenium can slow down neurodegenerative processes (Zafar et al. 2003). Supplementation with micronutrients including selenium was revealed to improve symptoms of depression in older people (Gosney et al. 2008) and sodium selenite was suggested to be a promising compound for treatment of Alzheimer’s disease (van Eersel et al. 2010). However, some authors reported opposite opinions (Loef et al. 2011). Vinceti et al. (2010) hypothesized that dietary inorganic selenium may increase the risk of amyotrophic lateral sclerosis. Organic selenocompounds were studied in regard to their application in neurodegenerative diseases and the results were promising (Jauslin et al. 2002). Considering the possibility of the application of the studied compounds A and B as selenium-supplements, the outcomes of the present study show that although chain selenosemicarbazide significantly decreased lipid peroxidation level, it also caused impairment of antioxidant defence. 3-(2-chlorobenzoylamino-)-2-(*o*-tolylimino-)-4-methyl-4-selenazoline of cyclic structure seems to be a more promising agent for future studies since it did not decrease elements of antioxidant barrier and increased GPx activity. As selenium is a constituent of GPx it could point that the bioavailability of selenazoline is better than that of selenosemicarbazide. Concluding, further studies with use of selenazoline appear to be advisable to evaluate its possible application as a selenium-supplement. As sex differences regarding selenium in vertebrates were reported (Raman et al. 2012) subsequent studies should include female rats.

## Abbreviations

TAS: Total antioxidant status
SOD: Superoxide dismutase
GPx: Glutathione peroxidase
AA: Ascorbic acid
MDA: Malonyl dialdehyde
GSH: Reduced glutathione
